# Supplementary material for: Molecular Dynamics Simulation Analysis of JAK1 Initial Activation: Phosphorylation-Induced Conformational Dynamics and Domain Interactions
Source: Life (Basel). 2025 Aug 19;15(8):1316. doi: 10.3390/life15081316 (PMC12387218; doi:10.3390/life15081316)
Supplement: Supplementary file 1 [file life-15-01316-s001.zip › life-3774393-supplementary.pdf]

Supporting information

Molecular Dynamics Simulation Analysis of JAK1 initial Activation: Phosphorylation-Induced Conformational Dynamics and Domain Interactions

Xinyu Peng[1+], Kefu Liu[1+], Guodong Chen[2*], Shengjie Sun[1*]

[1]*Department of Biomedical Informatic, School of Life Sciences, Central South University, Changsha, China, 410083;*

[2]*Center for Medical Genetics, School of Life Sciences, Central South University, Changsha, China; 410083;*

*Cooresponding author： Guodong Chen: guodong.chen@csu.edu.cn

Shengjie Sun shengjieswen@163.com

[+] These authors contributed equally to this work

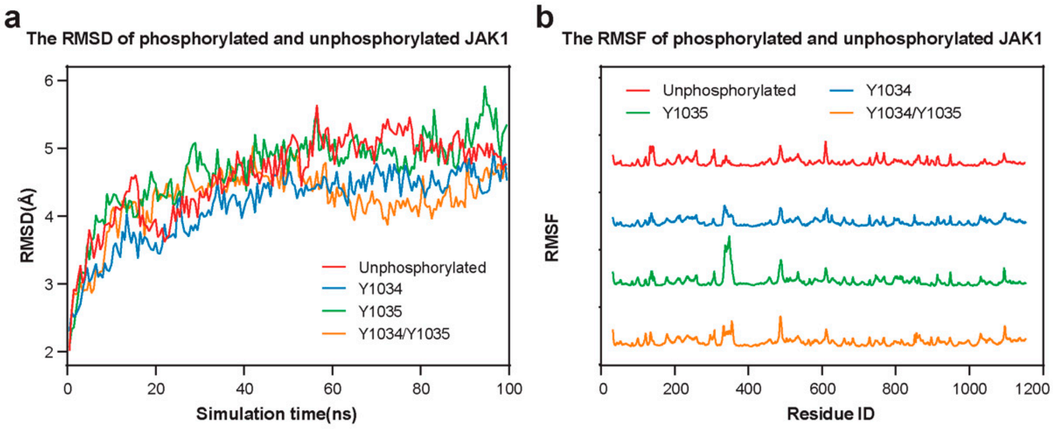

Figure S1: The conformational analysis of unphosphorylated and phosphorylated JAK1. a. The RMSD of the JAK1 monomer. b. The RMSF of JAK1 monomer.

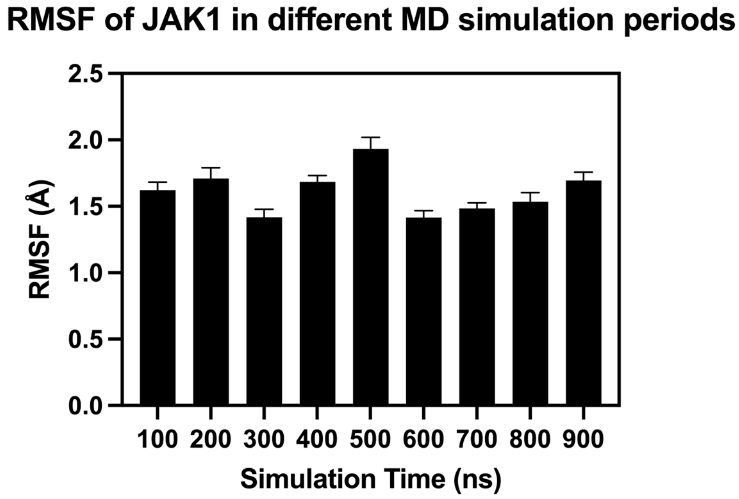

Figure S2: The average of residues RMSF in different periods.
